# A generalized gradient projection method based on a new working set for minimax optimization problems with inequality constraints

**DOI:** 10.1186/s13660-017-1321-3

**Published:** 2017-02-27

**Authors:** Guodong Ma, Yufeng Zhang, Meixing Liu

**Affiliations:** 1 School of Mathematics and Statistics, Guangxi Colleges and Universities Key Laboratory of Complex System Optimization and Big Data Processing, Yulin Normal University, Yulin, China; 2 College of Mathematics and Information Science, Guangxi University, Nanning, China

**Keywords:** 90C30, 49K35, 65K05, minimax optimization problems, inequality constraints, generalized gradient projection method, global and strong convergence

## Abstract

Combining the techniques of the working set identification and generalized gradient projection, we present a new generalized gradient projection algorithm for minimax optimization problems with inequality constraints. In this paper, we propose a new optimal identification function, from which we provide a new working set. At each iteration, the improved search direction is generated by only one generalized gradient projection explicit formula, which is simple and could reduce the computational cost. Under some mild assumptions, the algorithm possesses the global and strong convergence. Finally, the numerical results show that the proposed algorithm is promising.

## Introduction

Minimax problem is an important class of nonsmooth optimization, since it has a broad application background. Numerous models in optimal control [1, 2], engineering problem [3], portfolio optimization [4] and many other situations [5] can be formulated as the following minimax optimization problems with inequality constraints:

1 $$ \min F(x),\quad \mbox{s.t. } f_{j}(x) \le 0,\quad j \in J, $$

where $F(x) = \max \{ f_{j}(x),j \in I\}$ with $I = \{ 1,2, \ldots,m\}$, $j \in J = \{ l + 1,l + 2, \ldots,l + m\}$, $f_{j}(x):R^{n} \to R$ ($j \in I \cup J$) are continuously differentiable functions. The objective function $F(x)$ is not necessarily differentiable, even when all $f_{j}(x)$, $j \in \{ I \cup J\} $ are differentiable. Obviously, the non-differentiablity of the objective function $F(x)$ is a main challenge for solving minimax problem, as the classical smooth methods cannot be applied directly. Over the past few decades, the minimax problem has attracted more and more researchers’ attention and many algorithms have been developed, which can be grouped into three classes in general.

The first class of algorithms views the problem (1) as the constrained nonsmooth optimization problem, which can be solved directly by several classical nonsmooth methods, such as subgradient methods, bundle methods, and cutting plane methods, Refs. [6–9]. The algorithms in [6–8] have a shortcoming that it is difficult to improve the numerical results. However, we have observed in [9] that a feasible descent bundle method for solving inequality constrained minimax problems is proposed, by using the subgradients of functions, the idea of bundle method and the technique of partial cutting planes model, to generate the new cutting plane and aggregate the subgradients in the bundle, so the difficulty of numerical calculation and storage is overcome.

The second one is the entropy function method [10–13]. First, it transforms the objective function minimax problem into a smooth function with parameters. Then the objective function is approximated by a parametric and smooth function. For example, the parametric and smooth function with the parameter *p*

$$F_{p}(x) = \frac{1}{p}\ln \Biggl\{ \sum _{i = 1}^{l} \exp \bigl[pf_{i}(x)\bigr] \Biggr\} . $$

Since $0 \le F_{p}(x) - F(x) \le \frac{1}{p}\ln l$, so $p \to \infty$, $F_{p}(x) \to F(x)$.

Due to the particular structure of the objective function, the third approach is that the problem (1) can be transformed into the following equivalent smooth constrained nonlinear programming by introducing an artificial variable *z*:

$$\min_{(x,z) \in R^{n + 1}} z,\quad \mbox{s.t. }f_{i}(x) - z \le 0, \quad i \in I;\qquad f_{j}(x) \le 0,\quad j \in J. $$

Then, we can solve the above inequality constrained optimization by some well-established methods, such as the sequential quadratic programming type (SQP) methods [14–17], the sequential quadratically constrained quadratic programming type (SQCQP) methods [18, 19], the trust-region strategy [20, 21] and the interior-point method [22]. In [14], a SQP algorithm is proposed that incorporates the particular case of minimax problems, the global and local convergence is ensured. In order to improve the convergence properties and numerical performance, Jian and Zhu *et al*. developed improved SQP methods established in [15, 16] for solving unconstrained or constrained minimax problems, by means of solving one quadratic programming an improved direction is yielded and a second-order correction direction can also be at hand via one system of linear equations. Under mild conditions, we can ensure global and superlinear convergence. Jian *et al*. [17] developed the norm-Relaxed SQP method based on active set identification and new line search for constrained minimax problems, which the master direction and high-order correction direction are computed by solving a new type of norm-relaxed quadratic programming subproblem and a system of linear equations, respectively. Moreover, the step size is yielded by a new line search which combines the method of strongly sub-feasible direction with the penalty method.

Recently, several researchers have worked on the SQCQP, to solve unconstrained or constrained minimax problems. The authors provided the SQCQP method [18] and showed that their algorithm is faster than the SQP algorithm that is a well-known algorithm used for solving constrained minimization problems, which solved a subproblem that involves convex quadratic inequality constraints and a convex quadratic objective function. Jian *et al*. [19] also proposed the simple sequential quadratically constrained quadratic programming algorithm for smooth constrained optimization. Unlike the previous work, at each iteration, the main search direction is obtained by solving only one subprogram which is composed of a convex quadratic objective function and simple quadratic inequality constraints without the second derivatives of the constrained functions.

Although the SQP and SQCQP methods can effectively solve the minimax problem, this transformation may ignore the unique nature of the minimax problem and then increase the number of constraint functions. Moreover, these methods require the solution of one or two QP (QCQP) subproblems at each iteration, especially, some subproblems may be complex for the large-scale problems. In general, there are many cases where the subproblems cannot be solved easily, which will increase the amount of computations largely. Hence, (generalized) gradient projection method (GGPM) based on the Rosen gradient projection method [23] has been developed for solving inequality constrained optimization problems. The GGPM method has good properties that the search direction is only a gradient projection explicit formula and it has nice convergence and numerical results for middle-small-scale problems. These good natures cause widespread concern of many scholars [24–26]. In [25], Chapter II, there are more systematic and detailed study about generalized gradient projection algorithm for inequality constrained smooth optimization.

It is well established in the literature that, when the number of constraints is very large, the active set identification technique can improve the local convergence behavior and decrease the computation cost of the algorithms of nonlinear programming and minimax problems. An earlier study of the active set identification technique can be found in [27]. Many satisfactory results on the general nonlinear case were studied, *e.g.*, [28, 29]. Facchinei *et al*. [28] described a technique based on the algebraic representation of the constraint set, which identifies active constraints in a neighborhood of a solution. The extension to constrained minimax problems was also first presented in [30] without strict complementarity and linear independence. Moreover, the identification technique of active constraints for constrained minimax problems can be more suitable for infeasible algorithms, such as the strongly sub-feasible direction method and the penalty function method.

Despite GGPM’s importance and usefulness, there are no GGPM type method that are applied to solve minimax problems with inequality constraints. The aim of this paper is to propose such an algorithm, analyze its convergence properties, and report its numerical performance. Motivated by [26, 30], in this paper, we propose a generalized gradient projection algorithm directly on $R^{n}$ with a new working set for the problem (1). The characteristics of our proposed algorithm can be summarized as follows:

1. We propose a new optimal identification function for the stationary point, from which we provide a new working set.
2. The search direction is generated by only one generalized gradient projection explicit formula, which is simple and could reduce the computational cost.
3. Under some mild assumptions, the algorithm possesses the global and strong convergence.

The paper is organized as follows. The next section describes the algorithm. Section 3 discusses the convergence analysis. Section 4 contains numerical results. Finally, some conclusions are drawn in Section 5.

## Description of algorithm

In this section, for the sake of simplicity, we introduce and use the following notations for the problem (1) in this paper:

2 $$\begin{aligned}& X: = \bigl\{ x \in R^{n}: f_{j}(x) \le 0, j \in J\bigr\} , \end{aligned}$$

3 $$\begin{aligned}& I(x): = \bigl\{ i \in I:f_{i}(x) = F(x)\bigr\} ,\qquad J(x): = \bigl\{ j \in J:f_{j}(x) = 0\bigr\} . \end{aligned}$$

According to the analysis of other projection algorithms, we need the following linear independence assumption.

### Assumption A1

The functions $f_{j}(x)$ ($j \in I \cup J$) are all first order continuously differentiable, and there exists an index $l_{x} \in I(x)$ for each $x \in X$ such that the gradient vectors $\{ \nabla f_{i}(x) - \nabla f_{l_{x}}(x), i \in I(x)\backslash \{ l_{x}\};\nabla f_{j}(x),j \in J(x)\}$ are linearly independent.

### Remark 1

We can easily find that Assumption A1 is equivalent to Assumption A1-1:

### Assumption A1-1

The vectors $\{ \nabla f_{i}(x) - \nabla f_{t}(x), i \in I(x)\backslash \{ t\}; \nabla f_{j}(x),j \in J(x)\} $ are linearly independent for arbitrarily $t \in I(x)$.

For a given point $x^{k} $ and the parameter $\varepsilon \ge 0$, we use the following approximate active set in this paper:

4 $$ \textstyle\begin{cases} I_{k} = \{ i \in I: - \tilde{\varrho}_{k} \le f_{i}(x^{k}) - F(x^{k}) \le 0\}, \\ J_{k} = \{ j \in J: - \tilde{\varrho}_{k} \le f_{j}(x^{k}) \le 0\}, \end{cases} $$

where $\tilde{\varrho}_{k}$ is taken by

5 $$ \tilde{\varrho}_{0} = \varepsilon,\qquad \tilde{\varrho}_{k} = \min \{ \varepsilon,\varrho_{k - 1}\},\quad k \ge 1, $$

$\varrho_{k - 1} \ge 0$ is the identification function corresponding to the last iteration point $x^{k - 1} $ and it can be calculated by (17). At each iteration, only $\varrho_{k}$ need to be computed, since $\varrho_{k - 1} $ has already been calculated at the previous iteration, so the total computing capacity is not increase at each iteration.

For the current iteration point $x^{k}$, for convenience of statement, we define

$$\begin{aligned}& F^{k}: = F\bigl(x^{k}\bigr),\qquad f_{i}^{k}: = f_{i}\bigl(x^{k}\bigr),\qquad g_{i}^{k}: = \nabla f_{i}\bigl(x^{k}\bigr),\quad i \in I \cup J, \\& l_{k} \in I\bigl(x^{k}\bigr),\qquad I_{k}^{0}: = I_{k}\backslash \{ l_{k}\},\qquad L_{k}: = I_{k}^{0} \cup J_{k}: = (I_{k} \cup J_{k})\backslash \{ l_{k}\}, \end{aligned}$$

where $l_{k} \in I(x^{k})$. In theory, the index $l_{k}$ can be any component of the active set $I(x^{k})$ of Algorithm A, and all the theoretical analyses are the same. But we set $l_{k} = l_{x^{k}} =:\min \{ i:i \in I(x^{k})\} $ for convenience. We can easily find that $I(x^{k}) \subseteq I_{k}$, $J(x^{k}) \subseteq J_{k}$. Therefore, for the feasible point $x^{k}$ of the problem (1), its stationary point (optimality conditions) can be described as

6 $$ \textstyle\begin{cases} \sum_{i \in I_{k}}\lambda_{i}^{k}g_{i}^{k} + \sum_{j \in J_{k}}\lambda_{j}^{k}g_{j}^{k} = 0,\sum_{i \in I_{k}}\lambda_{i}^{k} = 1, \\ \lambda_{i}^{k} \ge 0,\lambda_{i}^{k}(f_{i}^{k} - F^{k}) = 0,i \in I_{k};\lambda_{j}^{k} \ge 0,\lambda_{j}^{k}f_{j}^{k} = 0,j \in J_{k}. \end{cases} $$

The foregoing conditions can be rewritten in the following equivalent form:

7 $$ \textstyle\begin{cases} \sum_{i \in I_{k}^{0}}\lambda_{i}^{k}(g_{i}^{k} - g_{l_{k}}^{k}) + \sum_{j \in J_{k}}\lambda_{j}^{k}g_{j}^{k} = - g_{l_{k}}^{k}, \\ \lambda_{i}^{k} \ge 0,\lambda_{i}^{k}(f_{i}^{k} - F^{k}) = 0,i \in I_{k}^{0};\lambda_{j}^{k} \ge 0,\lambda_{j}^{k}f_{j}^{k} = 0,j \in J_{k}, \\ \lambda_{l_{k}}^{k} = 1 - \sum_{i \in I_{k}^{0}}\lambda_{i}^{k} \ge 0. \end{cases} $$

We define a generalized gradient projection matrix to test whether the current iteration point $x^{k}$ satisfies (6)

8 $$ P_{k}: = E_{n} - N_{k}Q_{k}, $$

where $E_{n}$ is an *n*th-order identity matrix.

9 $$\begin{aligned}& N_{k}: = \bigl(g_{i}^{k} - g_{l_{k}}^{k},i \in I_{k}^{0};g_{j}^{k},j \in J_{k}\bigr),\qquad Q_{k}: = \bigl(N_{k}^{T}N_{k} + D_{k}\bigr)^{ - 1}N_{k}^{T}, \end{aligned}$$

10 $$\begin{aligned}& D_{k}: = \operatorname{diag}\bigl(D_{j}^{k},j \in L_{k}\bigr),\qquad D_{j}^{k}: = \textstyle\begin{cases} (F^{k} - f_{j}^{k})^{p},&j \in I_{k}^{0}; \\ ( - f_{j}^{k})^{p}, &j \in J_{k}. \end{cases}\displaystyle \end{aligned}$$

Suppose that ($x^{k},\lambda_{L_{k}}^{k}$) is a stationary point pair, from (7), it is not difficult to get

$$N_{k}^{T}N_{k}\lambda_{L_{k}}^{k} = - N_{k}^{T}g_{l_{k}}^{k},\qquad D_{k}\lambda_{L_{k}}^{k} = 0,\qquad \lambda_{L_{k}}^{k} \ge 0. $$

These further imply that

11 $$\begin{aligned}& \bigl(N_{k}^{T}N_{k} + D_{k}\bigr) \lambda_{L_{k}}^{k} = - N_{k}^{T}g_{l_{k}}^{k}, \qquad \lambda_{L_{k}}^{k} = - Q_{k}g_{l_{k}}^{k}, \end{aligned}$$

12 $$\begin{aligned}& P_{k}g_{l_{k}}^{k} = 0, \qquad \lambda_{l_{k}}^{k} \ge 0, \qquad D_{j}^{k}\lambda_{j}^{k} = 0,\qquad \lambda_{j}^{k} \ge 0,\quad j \in L_{k}. \end{aligned}$$

Based on the above analysis, we introduce the following optimal identification function for the stationary point:

13 $$ \rho_{k}: = \bigl\Vert P_{k}g_{l_{k}}^{k} \bigr\Vert ^{2} + \omega_{k} + \bar{\omega}_{k}^{2}, $$

where

14 $$\begin{aligned}& \omega_{k}: = \sum_{j \in L_{k}}\max \bigl\{ - \mu_{j}^{k},\mu_{j}^{k}D_{j}^{k} \bigr\} ,\qquad \bar{\omega}_{k}: = \max \bigl\{ - \mu_{l_{k}}^{k},0 \bigr\} , \end{aligned}$$

15 $$\begin{aligned}& \mu_{L_{k}}^{k}: = - Q_{k}g_{l_{k}}^{k},\qquad \mu_{l_{k}}^{k}: = 1 - \sum_{i \in I_{k}^{0}} \mu_{i}^{k}. \end{aligned}$$

Before describing our algorithm, one has the following lemma.

### Lemma 1

*Suppose that Assumption* A1 *holds*. *Then*

(i) *the matrix* $N_{k}^{T}N_{k} + D_{k}$ *is nonsingular and positive definite*. *Suppose* $x^{k} \to x^{*}$, $N_{k} \to N_{ *}$, $D_{k} \to D_{ *}$, *then the matrix* $N_{ *}^{T}N_{ *} + D_{ *}$ *is also nonsingular and positive definite*;
(ii) $N_{k}^{T}P_{k} = D_{k}Q_{k}$, $N_{k}^{T}Q_{k}^{T} = E_{\vert L_{k}\vert } - D_{k}(N_{k}^{T}N_{k} + D_{k})^{ - 1}$;
(iii) $(g_{l_{k}}^{k})^{T}P_{k}g_{l_{k}}^{k} = \Vert P_{k}g_{l_{k}}^{k}\Vert ^{2} + \sum_{j \in L_{k}}(\mu_{j}^{k})^{2}D_{j}^{k}$;
(iv) $\rho_{k} = 0$ *if and only if* $x^{k}$ *is a stationary point of the problem* (1).

### Proof

Under Assumption A1, it is shown that the matrix $N_{k}^{T}N_{k} + D_{k}$ is nonsingular by [25], Theorem 1.1.9. Then, we can prove that the matrix $N_{ *}^{T}N_{ *} + D_{ *}$ is also definite similar to [25], Lemma 2.2.2. The conclusions (ii) and (iii) can obtained similar to [25], Theorem 1.1.9. Next, we prove the conclusion (iv) in detail below.

(iv) If $\rho_{k} = 0$, combining the equations (13), (8), and (15), we obtain

$$0 = P_{k}g_{l_{k}}^{k} = g_{l_{k}}^{k} - N_{k}Q_{k}g_{l_{k}}^{k} = g_{l_{k}}^{k} + N_{k}\mu_{L_{k}}^{k}. $$

We have $\max \{ - \mu_{j}^{k},\mu_{j}^{k}D_{j}\} = 0$ by $\omega_{k} = 0$, which follows by $\mu_{j}^{k} \ge 0$, $\mu_{j}^{k}D_{j}^{k} = 0$, $\forall j \in L_{k} $ and it is not difficult to get $\mu_{l_{k}}^{k} \ge 0$ by $\bar{\omega}_{k} = 0$. So we have

$$\textstyle\begin{cases} \sum_{i \in I_{k}}\mu_{i}^{k}g_{i}^{k} + \sum_{j \in J_{k}}\mu_{j}^{k}g_{j}^{k} = 0, \sum_{i \in I_{k}}\mu_{i}^{k} = 1, \\ \mu_{i}^{k} \ge 0, \mu_{i}^{k}(f_{i}^{k} - F^{k}) = 0, i \in I_{k};\mu_{j}^{k} \ge 0, \mu_{j}^{k}f_{j}^{k} = 0, j \in J_{k}. \end{cases} $$

Therefore, $x^{k} $ is a stationary point of the problem (1) with the multiplier $(\mu_{I_{k} \cup J_{k}}^{k}, 0_{(I \cup J)\backslash (I_{k} \cup J_{k})})$.

Conversely, if $x^{k} $ is a stationary point of the problem (1) with the multiplier vector $\lambda^{k}$, then from the (6)-(15) one knows that $\mu_{L_{k}}^{k} = \lambda_{L_{k}}^{k}$, and $\rho_{k} = 0$. □

The above results show that the current iteration point $x^{k}$ is a stationary point of the problem (1) if and only if $\rho_{k} = 0$, that is, $\rho_{k} $ is optimal identification function. In case of $\rho_{k} > 0$, together with $I_{k}$ and $J_{k}$, we compute the search direction, which is motivated by the generalized gradient projection technique in [25], Chapter II, and [26]

16 $$ d^{k} = \rho_{k}^{\xi} \bigl\{ - P_{k}g_{l_{k}}^{k} + Q_{k}^{T}v_{L_{k}}^{k} \bigr\} - \varrho_{k}Q_{k}^{T}e^{k}, $$

where the parameter $\xi > 0$, $e^{k} = (1,1, \ldots,1)^{T} \in R^{\vert L_{k}\vert }$,

17 $$ \varrho_{k} = \frac{\rho_{k}^{1 + \xi}}{1 + \Vert \mu_{L_{k}}^{k}\Vert _{1}}, $$

the vector $v_{L_{k}}^{k} = (v_{j}^{k}, j \in L_{k})$ with

18 $$ v_{j}^{k} = \textstyle\begin{cases} \bar{\omega}_{k} - 1,& \mbox{if }\mu_{j}^{k} < 0,j \in I_{k}^{0}; \\ \bar{\omega}_{k} + D_{j}^{k},&\mbox{if }\mu_{j}^{k} \ge 0,j \in I_{k}^{0}, \end{cases}\displaystyle \qquad v_{j}^{k} = \textstyle\begin{cases} - 1,&\mbox{if }\mu_{j}^{k} < 0,j \in J_{k}; \\ D_{j}^{k},&\mbox{if }\mu_{j}^{k} \ge 0,j \in J_{k}. \end{cases} $$

The following lemma further describes the important characteristics of the search direction, which is feasible and descent.

### Lemma 2

*Suppose that Assumption* A1 *holds*. *Then*

(i) $(g_{l_{k}}^{k})^{T}d^{k} \le - \rho_{k}^{\xi} \bar{\omega}_{k} - \varrho_{k}$;
(ii) $(g_{j}^{k})^{T}d^{k} \le - \varrho_{k}$, $\forall j \in (I(x^{k})\backslash \{ l_{k}\} ) \cup J(x^{k})$;
(iii) $F'(x^{k};d^{k}) \le - \varrho_{k}$, *where* $F'(x^{k};d^{k})$ *is the directional derivative of* $F(x)$ *at the point* $x^{k}$ *along the direction* $d^{k}$.

### Proof

(i) First, by (16), together with Lemma 1(iii), (15), (18) and (14), we obtain

$$\begin{aligned} \bigl(g_{l_{k}}^{k}\bigr)^{T}d^{k} &= \rho_{k}^{\xi} \bigl\{ - \bigl(g_{l_{k}}^{k} \bigr)^{T}P_{k}g_{l_{k}}^{k} + \bigl(Q_{k}g_{l_{k}}^{k}\bigr)^{T}v_{L_{k}}^{k} \bigr\} - \varrho_{k}\bigl(Q_{k}g_{l_{k}}^{k} \bigr)^{T}e^{k} \\ &= \rho_{k}^{\xi} \biggl\{ - \bigl\Vert P_{k}g_{l_{k}}^{k}\bigr\Vert ^{2} - \sum _{j \in L_{k}}\bigl(\mu_{j}^{k} \bigr)^{2}D_{j}^{k} - \bigl(\mu_{L_{k}}^{k} \bigr)^{T}v_{L_{k}}^{k}\biggr\} + \varrho_{k} \bigl(\mu_{L_{k}}^{k}\bigr)^{T}e^{k} \\ &\le \rho_{k}^{\xi} \biggl\{ - \bigl\Vert P_{k}g_{l_{k}}^{k}\bigr\Vert ^{2} - \sum _{\mu_{i}^{k} < 0,i \in I_{k}^{0}}\mu_{i}^{k}(\bar{ \omega}_{k} - 1) - \sum_{\mu_{i}^{k} \ge 0,i \in I_{k}^{0}} \mu_{i}^{k}\bigl(\bar{\omega}_{k} + D_{i}^{k}\bigr) \\ &\quad {}- \sum_{\mu_{j}^{k} < 0,j \in J_{k}}\bigl( - \mu_{j}^{k} \bigr) - \sum_{\mu_{j}^{k} \ge 0,j \in J_{k}}\mu_{j}^{k}D_{j}^{k} \biggr\} + \varrho_{k}\bigl(\mu_{L_{k}}^{k} \bigr)^{T}e^{k} \\ &= \rho_{k}^{\xi} \biggl\{ - \bigl\Vert P_{k}g_{l_{k}}^{k}\bigr\Vert ^{2} - \sum _{\mu_{j}^{k} < 0,j \in L_{k}}\bigl( - \mu_{j}^{k}\bigr) - \sum_{\mu_{j}^{k} \ge 0,j \in L_{k}}\mu_{j}^{k}D_{j}^{k} - \bar{\omega}_{k}\sum_{i \in I_{k}^{0}} \mu_{i}^{k}\biggr\} + \varrho_{k}\bigl( \mu_{L_{k}}^{k}\bigr)^{T}e^{k} \\ &= \rho_{k}^{\xi} \biggl\{ - \bigl\Vert P_{k}g_{l_{k}}^{k}\bigr\Vert ^{2} - \omega_{k} - \bar{\omega}_{k}\sum _{i \in I_{k}^{0}}\mu_{i}^{k}\biggr\} + \varrho_{k}\bigl(\mu_{L_{k}}^{k}\bigr)^{T}e^{k}. \end{aligned} $$

In addition, based on the definition of $\mu_{l_{k}}^{k}$, it immediately follows $\bar{\omega}_{k}\sum_{i \in I_{k}^{0}}\mu_{i}^{k} = \bar{\omega}_{k} + \bar{\omega}_{k}( - \mu_{l_{k}}^{k})$. If $\mu_{l_{k}}^{k} \ge 0$, we have $\bar{\omega}_{k} = 0$, $\bar{\omega}_{k}( - \mu_{l_{k}}^{k}) = 0 = \bar{\omega}_{k}^{2}$. On the other hand $\mu_{l_{k}}^{k} < 0$, then $\bar{\omega}_{k} = - \mu_{l_{k}}^{k}$, $\bar{\omega}_{k}( - \mu_{l_{k}}^{k}) = \bar{\omega}_{k}^{2}$. Thus, $\bar{\omega}_{k}\sum_{i \in I_{k}^{0}}\mu_{i}^{k} = \bar{\omega}_{k} + \bar{\omega}_{k}^{2} $ is always true. We have from (13) and (17)

19 $$ \begin{aligned}[b] \bigl(g_{l_{k}}^{k} \bigr)^{T}d^{k} &\le \rho_{k}^{\xi} \bigl\{ - \bigl\Vert P_{k}g_{l_{k}}^{k}\bigr\Vert ^{2} - \omega_{k} - \bar{\omega}_{k} - \bar{\omega}_{k}^{2} \bigr\} + \varrho_{k}\bigl(\mu_{L_{k}}^{k} \bigr)^{T}e^{k} \\ &= \rho_{k}^{\xi} ( - \rho_{k} - \bar{ \omega}_{k}) + \varrho_{k}\bigl(\mu_{L_{k}}^{k} \bigr)^{T}e^{k} \\ &\le - \rho_{k}^{\xi} \bar{\omega}_{k} - \rho_{k}^{1 + \xi} + \varrho_{k}\bigl\Vert \mu_{L_{k}}^{k}\bigr\Vert _{1} \\ &= - \rho_{k}^{\xi} \bar{\omega}_{k} - \varrho_{k}. \end{aligned} $$

(ii) From Lemma 1(ii) and (16), we obtain

20 $$ \begin{aligned}[b] N_{k}^{T}d^{k} &= \rho_{k}^{\xi} \bigl\{ - N_{k}^{T}P_{k}g_{l_{k}}^{k} + N_{k}^{T}Q_{k}^{T}v_{L_{k}}^{k} \bigr\} - \varrho_{k}N_{k}^{T}Q_{k}^{T}e^{k} \\ &= \rho_{k}^{\xi} \bigl\{ - D_{k}Q_{k}g_{l_{k}}^{k} + v_{L_{k}}^{k} - D_{k}\bigl(N_{k}^{T}N_{k} + D_{k}\bigr)^{ - 1}v_{L_{k}}^{k}\bigr\} \\ &\quad {}- \varrho_{k}\bigl\{ E_{\vert L_{k}\vert } - D_{k} \bigl(N_{k}^{T}N_{k} + D_{k} \bigr)^{ - 1}\bigr\} e^{k}. \end{aligned} $$

Then we discuss the following two cases, respectively.

*Case 1.* For $i \in (I(x^{k})\backslash \{ l_{k}\} ) \subseteq I_{k}^{0}$, it follows that $D_{j}^{k} = 0$. From (20), we have $(g_{i}^{k} - g_{l_{k}}^{k})^{T}d^{k} = \rho_{k}^{\xi} v_{i}^{k} - \varrho_{k}$. Then, combined (18) with conclusion (i), we have

$$\bigl(g_{i}^{k}\bigr)^{T}d^{k} = \bigl(g_{l_{k}}^{k}\bigr)^{T}d^{k} + \rho_{k}^{\xi} v_{i}^{k} - \varrho_{k} \le - \varrho_{k} - \rho_{k}^{\xi} \bar{\omega}_{k} + \rho_{k}^{\xi} \bar{ \omega}_{k} - \varrho_{k} = - 2\varrho_{k} \le - \varrho_{k}. $$

*Case 2.* For $j \in J(x^{k}) \subseteq J_{k}$, $D_{j}^{k} = 0 $ holds. It follows from (20) and (18) that

$$\bigl(g_{j}^{k}\bigr)^{T}d^{k} = \rho_{k}^{\xi} v_{j}^{k} - \varrho_{k} \le - \varrho_{k}. $$

By summarizing the above discussion, the conclusion (ii) holds.

(iii) Since $F'(x^{k};d^{k}) = \max \{ (g_{i}^{k})^{T}d^{k}, i \in I(x^{k})\}$, we have $F'(x^{k};d^{k}) \le - \varrho_{k}$ from the conclusions (i) and (ii). □

Based on the improved direction $d^{k}$ defined by (16) and analysed above, we are now ready to describe the steps of our algorithm as follows.

### Algorithm A

*Step 0.* Choose an initial feasible point $x^{0} \in X$ and parameters: $\alpha,\beta \in (0,1)$, $\varepsilon > 0$, $p \ge 1$, $\xi > 0$. Let $k: = 0$.

*Step 1.* For the current iteration point $x^{k}$, generate the working set $I_{k}$, $J_{k}$ by (4) and (5), calculate the projection matrix $P_{k}$, the optimal identification function values $\rho_{k}$ and $\varrho_{k}$ by (8), (13)-(15) and (17). If $\rho_{k} = 0$, then $x^{k} $ is a stationary point of the problem (1), stop. Otherwise, go to Step 2.

*Step 2.* Obtain the search direction $d^{k}$ by (16)-(18).

*Step 3.* Compute the step size $t_{k}$, which is the maximum *t* of the sequence $\{ 1,\beta,\beta^{2}, \ldots \}$ satisfying

21 $$\begin{aligned}& F\bigl(x^{k} + td^{k}\bigr) \le F^{k} - \alpha t \varrho_{k}, \end{aligned}$$

22 $$\begin{aligned}& f_{j}\bigl(x^{k} + td^{k}\bigr) \le 0,\quad j \in J. \end{aligned}$$

*Step 4.* Let $x^{k + 1} = x^{k} + t_{k}d^{k}$, $k: = k + 1$, and go back to Step 1.

### Remark 2

The inequality (21) is equivalent to $f_{i}(x^{k} + td^{k}) \le F^{k} - \alpha t\varrho_{k}$, $i \in I$.

Note that Lemma 2, we get $F'(x^{k};d^{k}) \le - \varrho_{k} < 0 $ and $(g_{j}^{k})^{T}d^{k} \le - \varrho_{k}$, $\forall j \in J(x^{k})$, so it is easy to know that (21) and (22) hold for $t > 0$ small enough, then Algorithm A is well defined.

## Convergence analysis

In this section, we will analyze the global and strong convergence of Algorithm A. If Algorithm A stops at $x^{k}$ in a finite number of iterations, then $\rho_{k} = 0$. From Lemma 1(iv), we know that $x^{k}$ is a stationary point of the problem (1). Next, we assume that Algorithm A yields an infinite iteration sequence $\{ x^{k}\}$ of points, and prove that any accumulation point $x^{*}$ of $\{ x^{k}\}$ is a stationary point for the problem (1) under some mild conditions including the following assumption.

### Assumption A2

The sequence $\{ x^{k}\}$ generated by Algorithm A is bounded.

### Lemma 3

*Suppose that Assumptions* A1 *and* A2 *hold*. *Then*

(i) *there exists a positive number* *c* *such that* $\Vert d^{k}\Vert \le c\rho_{k}^{\xi}$;
(ii) $\lim_{k \to \infty} \Vert x^{k + 1} - x^{k}\Vert = 0$.

### Proof

(i) Because of Assumption A2 and Lemma 1(i), it is easy to get the total sequences $\{ P_{k}\}_{k = 1}^{\infty}$ and $\{ Q_{k}\}_{k = 1}^{\infty}$ of matrices are bounded. Furthermore, from (15) and (18), we know that $\{ \mu_{L_{k}}^{k}\}$ and $\{ v_{L_{k}}^{k}\}$ are bounded. This implies that $\Vert d^{k}\Vert \le c\rho_{k}^{\xi}$.

(ii) In view of $\{ F(x^{k})\}$ is decreasing and bounded, so $\lim_{k \to \infty} F(x^{k}) = F(x^{*})$. Then, combining (17) with the definition of $\varrho_{k}$, one has $\lim_{k \to \infty} t_{k}\rho_{k}^{1 + \xi} = 0$. Taking into account the parameter $\xi > 0$, it follows that

$$\lim_{k \to \infty} \bigl\Vert x^{k + 1} - x^{k} \bigr\Vert = \lim_{k \to \infty} t_{k}\bigl\Vert d^{k}\bigr\Vert \le \lim_{k \to \infty} ct_{k} \rho_{k}^{\xi} = c\lim_{k \to \infty} \bigl[ \bigl(t_{k}\rho_{k}^{1 + \xi} \bigr)^{\xi} t_{k}\bigr]^{\frac{1}{1 + \xi}} = 0. $$

 □

Based on the lemma above, we can present the global convergence of our algorithm.

### Theorem 1

*Suppose that Assumptions* A1 *and* A2 *hold*. *Then Algorithm* A *generates an infinite sequence* $\{ x^{k}\}$ *of points such that each accumulation* $x^{*}$ *of* $\{ x^{k}\}$ *is a stationary point of the problem* (1).

### Proof

If the infinite iteration index set $\mathcal{K}$ satisfies $\lim_{k \in \mathcal{K}}x^{k} = x^{ *}$, it follows that $\lim_{k \in \mathcal{K}}x^{k - 1} = x^{ *}$ from Lemma 3(ii). Noting that $I_{k}$, $J_{k}$ and $l_{k}$ are finite, we suppose $\forall k \in \mathcal{K}$ (choosing a subsequence of $\mathcal{K}$ when necessary) satisfies

23 $$\begin{aligned}& I_{k} \equiv I_{ *},\qquad J_{k} \equiv J_{ *},\qquad l_{k} \equiv l_{ *},\qquad I_{k}^{0} \equiv I_{ *}^{0}: = \{ I_{ *} \} \backslash l_{ *},\qquad L_{k} \equiv L_{ *}: = I_{ *}^{0} \cup J_{ *}, \end{aligned}$$

24 $$\begin{aligned}& \begin{aligned} &I_{k - 1} \equiv \bar{I}_{ *},\qquad J_{k - 1} \equiv \bar{J}_{ *},\qquad l_{k - 1} \equiv \bar{l}_{ *},\\ &I_{k - 1}^{0} \equiv \bar{I}_{ *}^{0}: = \{ \bar{I}_{ *} \} \backslash \bar{l}_{ *},\qquad L_{k - 1} \equiv \bar{L}_{ *}: = \bar{I}_{ *}^{0} \cup \bar{J}_{ *}. \end{aligned} \end{aligned}$$

Define

25 $$\begin{aligned}& D_{j}^{ *} = \textstyle\begin{cases} (F(x^{ *} ) - f_{j}(x^{ *} ))^{p}, &j \in I_{ *} \backslash \{ l_{ *} \}; \\ ( - f_{j}(x^{ *} ))^{p}, &j \in J_{ *}, \end{cases}\displaystyle \qquad D_{ *} = \operatorname{diag}\bigl(D_{j}^{ *},j \in L_{ *} \bigr), \end{aligned}$$

26 $$\begin{aligned}& N_{ *} = \bigl(\nabla f_{i}\bigl(x^{ *} \bigr) - \nabla f_{l_{ *}} \bigl(x^{ *} \bigr), i \in I_{ *}^{0}; \nabla f_{j}\bigl(x^{ *} \bigr),j \in J_{ *} \bigr),\qquad P_{ *} = E_{n} - N_{ *} Q_{ *}, \end{aligned}$$

27 $$\begin{aligned}& Q_{ *} = \bigl(N_{ *}^{T}N_{ *} + D_{ *} \bigr)^{ - 1}N_{ *}^{T}, \mu_{L_{ *}}^{ *} = \bigl(\mu_{j}^{ *},j \in L_{ *} \bigr) = - Q_{ *} \nabla f_{l_{ *}} \bigl(x^{ *} \bigr), \end{aligned}$$

28 $$\begin{aligned}& \omega_{ *} = \sum_{j \in L_{ *}} \max \bigl\{ - \mu_{j}^{ *}, \mu_{j}^{ *} D_{j}^{ *} \bigr\} ,\qquad\mu_{l_{ *}}^{ *} = 1 - \sum_{i \in I_{ *}^{0}}\mu_{i}^{ *},\qquad \bar{ \omega}_{ *} = \max \bigl\{ - \mu_{l_{ *}}^{ *},0\bigr\} , \end{aligned}$$

29 $$\begin{aligned}& \rho_{ *} = \Vert P_{ *} \nabla f_{l_{ *}} \bigl(x^{ *} \bigr)\Vert ^{2} + \omega_{ *} + \bar{ \omega}_{ *}^{2},\qquad \varrho_{ *} = \frac{\rho_{ *}^{1 + \xi}}{1 + \Vert \mu_{L_{ *}}^{ *} \Vert _{1}}. \end{aligned}$$

The above definitions are well defined by Lemma 1(i). Similarly, for $x^{ *}$, $\bar{I}_{ *}$, $\bar{J}_{ *}$ and $\bar{l}_{ *}$, we define $\bar{\rho}_{ *}$ and $\bar{\varrho}_{ *}$. In addition, the matrix $N_{ *}^{T}N_{ *} + D_{ *}$ is positive definite by Lemma 1(i). Therefore, the sequences $\{ v^{k}\}_{\mathcal{K}}$ and $\{ d^{k}\}_{\mathcal{K}} $ are bounded. Then, assume by contradiction that $x^{ *}$ is not the stationary point of the problem (1), we can easily get $\varrho_{ *} > 0 $ and $\bar{\varrho}_{ *} > 0$ similar to the analysis of Lemma 1(iv). Then, we have

$$\begin{aligned}& \varrho_{k} \ge 0.5\varrho_{ *},\qquad \varrho_{k - 1} \stackrel{\mathcal{K}}{\to}\bar{\varrho}_{ *}: = \frac{\bar{\rho}_{ *}}{1 + \Vert \bar{\mu}_{\bar{L}_{ *}}^{ *} \Vert } > 0,\\& \tilde{ \varrho}_{k} = \min \{ \varepsilon,\varrho_{k - 1}\} \stackrel{\mathcal{K}}{\to}\varrho_{ *}^{ \sim}: = \min \{ \varepsilon, \bar{\varrho}_{ *} \} > 0. \end{aligned}$$

Next, we will prove this theorem in two steps.

A. The step size sequence $\{ t_{k}\}_{k \in \mathcal{K}}$ is always bounded away from zero, that is, $\bar{t}: = \inf \{ t_{k}, k \in \mathcal{K}\} > 0$. We only need to show the inequalities (21) and (22) hold for $k \in \mathcal{K}$ large enough and $t > 0 $ sufficiently small.

A1. For $i \in I$, in this case, we have $i \notin I(x^{*})$ and $i \in I(x^{*})$ as two cases.

A11. Case $i \notin I(x^{*})$, that is $f_{i}(x^{ *} ) < F(x^{ *} )$. Taking into account the differentiability of $f_{i}(x)$ and the boundedness of $\{ d^{k}\}_{\mathcal{K}}$ and using Taylor expansion, we have

$$\begin{aligned} f_{i}\bigl(x^{k} + td^{k}\bigr) - F^{k} + \alpha t\varrho_{k}& = f_{i}^{k} + t\bigl(g_{i}^{k}\bigr)^{T}d^{k} + o(t) - F^{k} + \alpha t\varrho_{k} \\ &= f_{i}^{k} - F^{k} + O(t) \\ &\le 0.5\bigl(f_{i}\bigl(x^{ *} \bigr) - F \bigl(x^{ *} \bigr)\bigr) + O(t) \\ &\le 0. \end{aligned} $$

A12. Case $i \in I(x^{*})$, that is, $f_{i}(x^{*}) = F(x^{*})$. Note that $x^{k} \to x^{ *}$ and $\varrho_{ *}^{ \sim} > 0$, by (4) and (5), we know that $I(x^{ *} ) \subseteq I_{k} $ always holds for $k \in \mathcal{K}$ large enough. Hence, $i \in I_{k}$. In this case, we also have two cases, which are $i = l_{ *}$ and $i \ne l_{ *}$. Therefore, we discuss the two cases, respectively.

Assume $i = l_{ *}$, it follows by Lemma 2(i) that

30 $$ \bigl(g_{l_{ *}}^{k}\bigr)^{T}d^{k} \le - \rho_{k}^{\xi} \bar{\omega}_{k} - \varrho_{k} \le - \varrho_{k}. $$

Assume $i \ne l_{ *}$, $D_{i}^{k} = (F^{k} - f_{i}^{k})^{p} \stackrel{\mathcal{K}}{\to}(F(x^{ *} ) - f_{i}(x^{ *} ))^{p} = 0$. From Lemma 1(i), we have $(N_{k}^{T}N_{k} + D_{k})^{ - 1} \to (N_{ *}^{T}N_{ *} + D_{ *} )^{ - 1}$. Together with (20) we conclude that

$$\bigl(g_{i}^{k} - g_{l_{k}}^{k} \bigr)^{T}d^{k} = \rho_{k}^{\xi} v_{i}^{k} - \varrho_{k} + O\bigl(D_{i}^{k} \bigr). $$

In view of (18), $v_{i}^{k} \le \bar{\omega}_{k} + O(D_{i}^{k})$ holds. Combining (19) with $\varrho_{k} \to \varrho_{ *} > 0$, we have

31 $$ \bigl(g_{i}^{k}\bigr)^{T}d^{k} \le \rho_{k}^{\xi} \bar{\omega}_{k} + \bigl(g_{l_{k}}^{k}\bigr)^{T}d^{k} - \varrho_{k} + O\bigl(D_{i}^{k}\bigr) \le - 2 \varrho_{k} + O\bigl(D_{i}^{k}\bigr) \le - \varrho_{k}. $$

Thus, for $i \in I(x^{ *} )$, from (30) and (31), using Taylor expansion, we obtain

$$\begin{aligned} f_{i}\bigl(x^{k} + td^{k}\bigr) - F^{k} + \alpha t\varrho_{k} &\le f_{i}^{k} + t\bigl(g_{i}^{k}\bigr)^{T}d^{k} + o(t) - f_{i}^{k} + \alpha t\varrho_{k} \\ &\le - (1 - \alpha )t\varrho_{k} + o(t) \\ &\le - 0.5t(1 - \alpha )\varrho_{ *} + o(t) \\ &\le 0. \end{aligned} $$

According to the analysis of the above A11 and A12, it is sufficient to show that the inequality (21) is always satisfied for $k \in \mathcal{K}$ large enough and $t > 0$ sufficiently small.

A2. For $j \in J$, there are also $j \notin J(x^{*})$ and $j \in J(x^{*}) $ two cases.

A21. Case $j \notin J(x^{*})$, it follows that $f_{j}(x^{*}) < 0$. Taking into account the boundedness of $\{ d^{k}\}_{\mathcal{K}}$ and using Taylor expansion, we have

32 $$ f_{j}\bigl(x^{k} + td^{k}\bigr) = f_{j}^{k} + O(t) \le 0.5f_{j} \bigl(x^{*}\bigr) + O(t) \le 0. $$

A22. Case $j \in J(x^{*})$, that is, $f_{j}(x^{*}) = 0$. $j \in J_{k} $ follows in a similar analysis to A12, and we have $D_{j}^{k} = ( - f_{j}^{k})^{p} \stackrel{\mathcal{K}}{\to}0$. By recalling (20), $(g_{j}^{k})^{T}d^{k} = \rho_{k}^{\xi} v_{j}^{k} - \varrho_{k} + O(D_{j}^{k})$. Then using Taylor expansion and (18), we get

$$\begin{aligned} f_{j}\bigl(x^{k} + td^{k}\bigr) &= f_{j}^{k} + t\bigl(g_{j}^{k} \bigr)^{T}d^{k} + o(t) \\ &\le f_{j}^{k} - t\varrho_{k} + tO \bigl(D_{j}^{k}\bigr) + o(t) \\ &\le - 0.5t\varrho_{*} + o(t) \\ &\le 0. \end{aligned} $$

Thus, inequality (22) always holds for $k \in \mathcal{K}$ large enough and $t > 0$ sufficiently small. Hence, $\bar{t}: = \inf \{ t_{k}, k \in \mathcal{K}\} > 0$ holds.

B. Export contradiction by using $t_{k} \ge \bar{t} > 0$ ($k \in \mathcal{K}$). In view of (21), it is easy to know that the sequence $\{ F^{k}\}$ is monotone nonincreasing. Since $\lim_{k \in \mathcal{K}}F^{k} = F(x^{ *} )$, it implies $\lim_{k \to \infty} F^{k} = F(x^{ *} )$. This, together with (21) and $t_{k} \ge \bar{t}$, $\varrho_{k} \ge 0.5\varrho_{ *}$, we have

$$F\bigl(x^{ *} \bigr) = \lim_{k \in \mathcal{K}}F^{k + 1} \le \lim_{k \in \mathcal{K}}\bigl(F^{k} - \alpha t_{k} \varrho_{k}\bigr) \le \lim_{k \in \mathcal{K}}\bigl(F^{k} - 0.5\alpha \bar{t}\varrho_{ *} \bigr) = F\bigl(x^{ *} \bigr) - 0.5\alpha \bar{t}\varrho_{ *}, $$

which contradicts $\bar{t} > 0$ and $\varrho_{ *} > 0$. Therefore, $x^{*} $ is a stationary point for the problem (1), and the whole proof is completed. □

Subsequently, we further show that Algorithm A has the property of strong convergence.

### Theorem 2

*Suppose that Assumptions* A1 *and* A2 *hold*. *If the sequence* $\{ x^{k}\}$ *of points generated by Algorithm* A *possesses an isolated accumulation point* $x^{ *}$, *then* $\lim_{k \to \infty} x^{k} = x^{ *}$, *that is*, *Algorithm* A *is strongly convergent*.

### Proof

Since the sequence $\{ x^{k}\}$ of points generated by Algorithm A possesses an isolated accumulation point, together with $\lim_{k \to \infty} \Vert x^{k + 1} - x^{k}\Vert = 0$ (Lemma 3(ii)) and [25], Corollary 1.1.8, we immediately have $\lim_{k \to \infty} x^{k} = x^{ *}$. Finally, by $\lim_{k \to \infty} x^{k} = x^{ *}$ and Theorem 1, it follows that $x^{ *}$ is the stationary point of the problem (1). □

## Numerical experiments

In this section, in order to validate the efficiency of our proposed Algorithm A, some preliminary numerical experiments have been carried out. Test problems are divided into two groups. The first test group is made up of 10 problems (P1-P10), of which four small scale problems P1-P4 are taken from [25], which is the problem 7.3.28, 7.3.32, 7.3.31 and 7.3.29, respectively; and the other six middle-large-scale problems P5-P10 are from [31]. These six problems are composed of the corresponding objective functions and constraint functions in [31]. The second test group, we pick up six problems from [31] and compare the results of Algorithm A with the algorithm from [17] (called Algorithm B). In particular, $\mathrm{P}5=2.3+4.1(1)$ (which means the objective and constraints of the problem P11 are 2.3 and 4.1(1) in [31], respectively, and the same blew), $\mathrm{P}6=2.3+4.6(2)$, $\mathrm{P}7=2.1+4.6(2)$, $\mathrm{P}8=2.1+4.1(1)$, $\mathrm{P}9=2.5+4.6(2)$ ($n=200$), $\mathrm{P}10=2.9+4.1(1)$, $\mathrm{P}11=2.1+4.6(1)$, $\mathrm{P}12=2.5+4.6(2)$ ($n=50$), $\mathrm{P}13=2.9+4.6(2)$.

Algorithm A is coded in MATLAB R2010b, and on a PC computer with Windows 7, Intel(R) Pentium(R) CPU 2.4 GHz. During the numerical experiments, we set parameters $\alpha = 0.4$, $\beta = 0.4$, $\varepsilon = 7$, $p = 1$, $\xi = 0.2$. Execution is terminated if $\rho_{k} < 10^{ - 5}$ or $\mathrm{NI}\ge 150$.

The numerical results are listed in Tables 1 and 2. The following notations are used: ‘Prob.’: the test problem number; ‘n’: the dimensions of the variable *x*; ‘*l*‘: number of all component objective functions; ‘*m*’: number of constraint functions; ‘$x^{0}$’: initial feasible point; ‘Alg.’: algorithm; ‘A and B’: the algorithms of this paper and [17], respectively. ‘NI’: number of iterations; ‘NF’: number of all component functions evaluations in the objective; ‘NC’: number of constraints evaluations; ‘T’: CPU time (in seconds); ‘-’: the corresponding datum are not reported; ‘$F(x^{*})$’: final objective value.

**Table 1:** **Numerical results of Algorithm** **A**

Prob.	*n*/*l*/*m*	$x^{0}$	NI	NF	NC	$F(x^{*})$	T (s)
P1	2/3/2	$(1,2.4)^{T}$	15	115	33	1.952225	0.03
P2	2/3/2	$(0,1)^{T}$	31	44	63	2.000009	0.01
P3	4/4/3	$(0,0.9,0.9, - 1.5)^{T}$	28	143	89	−43.999992	0.01
P4	2/6/2	$(1,2.4)^{T}$	18	379	37	0.616433	0.02
P5	50/2/48	$(2, \ldots,2)^{T}$	85	551	4299	−69.296460	0.27
P6	50/2/48	$(0.5, \ldots,0.5)^{T}$	150	6061	17026	−56.502976	0.79
P7	100/2/98	$(0.5, \ldots,0.5)^{T}$	22	160	2157	0.000006	0.13
P8	100/100/98	$(1, \ldots,1)^{T}$	98	691	9605	0.500009	1.22
P9	200/3/199	$(0.5, \ldots,0.5)^{T}$	102	1087	22502	398.000010	2.72
P10	200/2/198	$(1, \ldots,1)^{T}$	150	456	29723	111.701918	3.92

**Table 2:** **Numerical comparisons for Algorithms** **A** **and B**

Prob.	*n*/*l*/*m*	$x^{0}$	Alg.	NI	NF	NC	$F(x^{*})$	T (s)
P5	50/2/48	$(1, \ldots,1)^{T}$	A	65	551	4299	−69.296460	0.27
			B	43	141	4178	−69.296456	4.53
P6	50/2/48	$(0.5, \ldots,0.5)^{T}$	A	150	6061	17026	−56.502976	0.79
			B	92	278	9065	56.580323	7.61
P8	50/2/48	$(1, \ldots,1)^{T}$	A	96	673	4609	0.500010	0.70
			B	9	949	887	0.500000	2.34
P11	50/2/49	$(0.4, \ldots,0.4)^{T}$	A	82	1804	4019	0.111121	0.66
			B	6	650	638	0.111111	2.17
P12	50/3/49	$(0.5, \ldots,0.5)^{T}$	A	24	202	1177	0.000001	0.09
			B	10	38	784	0	2.05
P13	50/3/49	$(0.5, \ldots,0.5)^{T}$	A	80	652	3969	98.000010	0.35
			B	147	631	14455	98.000001	10.94
Total				497				2.84
				307				29.64

From Table 1, we see that Algorithm A can generate approximately optimal solution for all the test problems. The search direction is generated by the generalized gradient projection explicit formula, and a new working set is used, which can reduce the scale of the generalized gradient projection, so the proposed Algorithm A is efficient.

In Table 2, we compare our Algorithm A with Algorithm B, and all the numerical results are tested by a same PC computer. The performance of the two algorithms is similar in terms of the approximate optimal objective value at the final iteration point, although the number of all component functions evaluations in the objective and constraints evaluations is few for Algorithm B, our proposed algorithm performs much better than Algorithm B relative to the cost of CPU running times.

In summary, through the numerical results in two tables and the analysis above, we can get that the proposed Algorithm A is promising for middle-small-scale minimax problem.

## Conclusions

Although the generalized gradient projection algorithms have good theoretical convergence and effectiveness in practice, their applications to minimax problems have not yet been investigated. In this paper, we present a new generalized gradient projection algorithm for minimax optimization problems with inequality constraints.

The main conclusions of this work:

1. A new approximation working set is presented.
2. Using the technique of generalized gradient projection, we construct a generalized gradient projection feasible decent direction.
3. Under mild assumptions, the algorithm is global and strong convergent.
4. Some preliminary numerical results show that the proposed algorithm performs efficiently.

## Results and discussion

In this work, a new generalized gradient projection algorithm for minimax optimization problems with inequality constraints is presented. As further work, we think the ideas can be extended to minimax optimization problems with equality and inequality constraints and other optimization problems.
